# Energy-absorption analyses of honeycomb-structured Al-alloy and nylon sheets using modified split Hopkinson pressure bar

**DOI:** 10.1038/s41598-023-49386-6

**Published:** 2023-12-18

**Authors:** Selim Kim, Minu Kim, Ki Jong Kim, Jae Min Lee, Hae-Won Cheong, Hyoung Seop Kim, Sunghak Lee

**Affiliations:** 1 Department of Materials Science and Engineering, Pohang University of Science and Technology, Pohang, 37673 Republic of Korea; 2 Agency for Defense Development, Daejeon, 34186 Republic of Korea; 3 The One Metal Inc., Ansan, 15599 Republic of Korea; 4 PGM R&D Institute, Hanwha Aerospace, Daejeon, 34101 Republic of Korea; 5 Graduate Institute of Ferrous & Eco Materials Technology, Pohang University of Science and Technology, Pohang, 37673 Republic of Korea

**Keywords:** Materials for devices, Structural materials

## Abstract

Thin cylindrical honeycomb-structured aluminum alloy and mono-cast (MC) nylon were studied as superior energy-absorbing materials compared to metallic foams. Their energy-absorbing performance was assessed using a modified split Hopkinson pressure bar (SHPB). Key parameters included maximum impact acceleration (*a*^*max*^) and its reduction ratio (compared to the none-specimen case). The lowest *a*^*max*^ reduction ratio was observed in bulk Al sheets without honeycomb cavities. As the cavity fraction increased up to 79% in honeycomb-structured Al specimens, the *a*^*max*^ reduction ratio improved due to broadened stress–time curves with a shallow-plateau shape. This made high-cavity-fraction Al specimens preferable for higher-energy absorption and lighter-weight buffering materials. In nylon specimens, the *a*^*max*^ reduction ratio increased until the fraction reached 52% due the softer and more deformable nature of the polymeric nylon. Thicker or rotated Al specimens also showed higher *a*^*max*^ reduction ratios due to sufficient and continuous energy absorption. The modified SHPB demonstrated effective energy-buffering concepts and provided insightful *a*^*max*^ interpretations, overcoming complexities in energy absorption analyses.

## Introduction

Metallic foams excel at absorbing compressive impact energy due to their three-dimensional load-bearing networks^1–3^. However, accurately evaluating their energy-absorbing performance is challenging as most impact energy dissipates rapidly when internal pores inside the foams close^4–6^. Controlling the size, volume fraction, and distribution of these pores is difficult using existing foam-making methods^7–9^. To overcome these limitations, this study proposes using thin cylindrical sheets made of aluminum alloy or mono-cast (MC) nylon with honeycomb structures as superior energy-absorbing materials compared to metallic foams. The honeycomb-structured sheets allow for controlled cavity parameters and easy scalability by stacking multiple sheets. However, detailed studies on the energy-absorbing performance mechanisms of these sheets are lacking.

Energy-absorbing materials widely used in military, automotive, aerospace, and civil-engineering fields require high specific strength, fracture toughness, and high impact resistance^10,11^. Evaluating their safety under impact loading environments like artillery firing, automotive collisions, and blast impacts is crucial. Yet, existing evaluation methods, such as full-scale artillery or gas-gun tests, have limitations in assessing the safety of energy-absorbing materials independently. Reliable evaluation methods are needed to improve energy-absorbing performance and assess safety effectively, but such methods have not been provided.

This study aims to evaluate the energy-absorbing performance of honeycomb-structured Al-alloy and MC nylon sheets using a modified split Hopkinson pressure bar (SHPB). Key parameters for evaluation include the maximum impact acceleration measured from stress–time curves of the modified SHPB and the reduction in acceleration caused by introducing the Al or nylon sheet. These results will be crucial in applying Al and nylon sheets as effective buffer materials in dynamically compressed artillery environments.

## Results

### Stress–time (*σ*–t) curves

**Figure 1:**
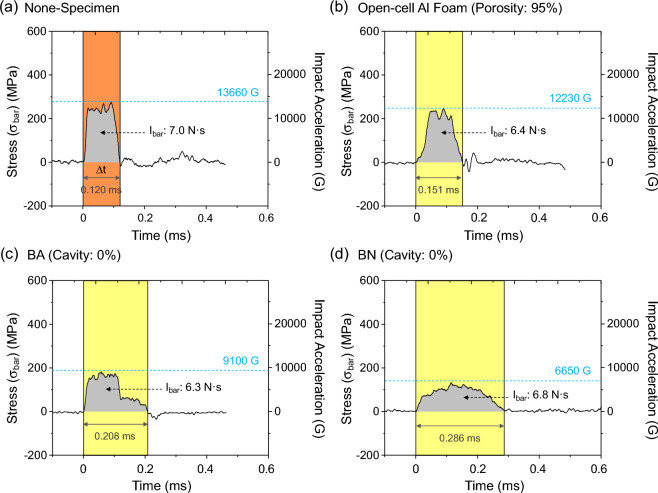
(**a**) Typical stress (*σ*, MPa)–time (t, millisecond (ms)) curve obtained by converting the voltage–time data recorded in the oscilloscope in the case where the test specimen is absent from the deceleration-measuring module (referred to as “none-specimen” case) under an air pressure of 0.3 MPa. (**b–d**) shows *σ*–t curves of the modules, inside which the 6-mm-thick open-cell Al foam, bulk BA specimen, or bulk BN specimen is placed between the transfer and incident bars.

A typical stress (*σ*, MPa) versus time (t, millisecond (ms)) curve was obtained by converting the voltage versus time data recorded in the oscilloscope at the air pressure of 0.3 MPa, as shown in Fig. 1a. This curve represents the case where the test specimen is absent from the deceleration-measuring module, referred to as the ‘none-specimen case’. In the modified SHPB setup, there are no transmitted or reflected waves^12^. The stress in the incident bar (*σ*_*bar*_) can be determined using the following equation:

$${\sigma }_{bar}=\sigma = \left(\frac{2}{{F}_{gage}}\cdot \frac{{E}_{bar}}{{V}_{in}}\right){V}_{out} ({\text{MPa}})$$

where *F*_*gage*_, *E*_*bar*_, *V*_*in*_, and *V*_*out*_ are the gage factor (3.22), elastic modulus of the incident bar made of a maraging steel (200 GPa)^13^, input voltage (24 V), and output voltage, respectively. The incident wave length (*Δt*) expressed as a time term is represented by a yellow-colored area. The *σ* increases rapidly to about 250 MPa, remains there for about 0.10 ms, and drops to produce a near-rectangular curve shape (Fig. 1a). This curve is almost identical to the incident wave or those of other conventional metallic foams or alloys obtained from the original SHPB setup^14,15^.

Figure 1b–d presents *σ*–t curves of the module with the 6-mm-thick open-cell Al foam, bulk BA, or bulk BN specimens. The *σ* of the Al foam specimen increases rapidly to 235 MPa, holds for 0.043 ms, and then decreases (Fig. 1b). The BA specimen shows a rapid rise to 170 MPa, stays for 0.170 ms, and drops in a two-step manner (Fig. 1c). The *σ*–t curve of the BN specimen has a typical up-and-down broad-peak shape (Fig. 1d). The curve height decreases in the order of Al foam, BA, and BN specimens, while *Δt* increases.

**Figure 2:**
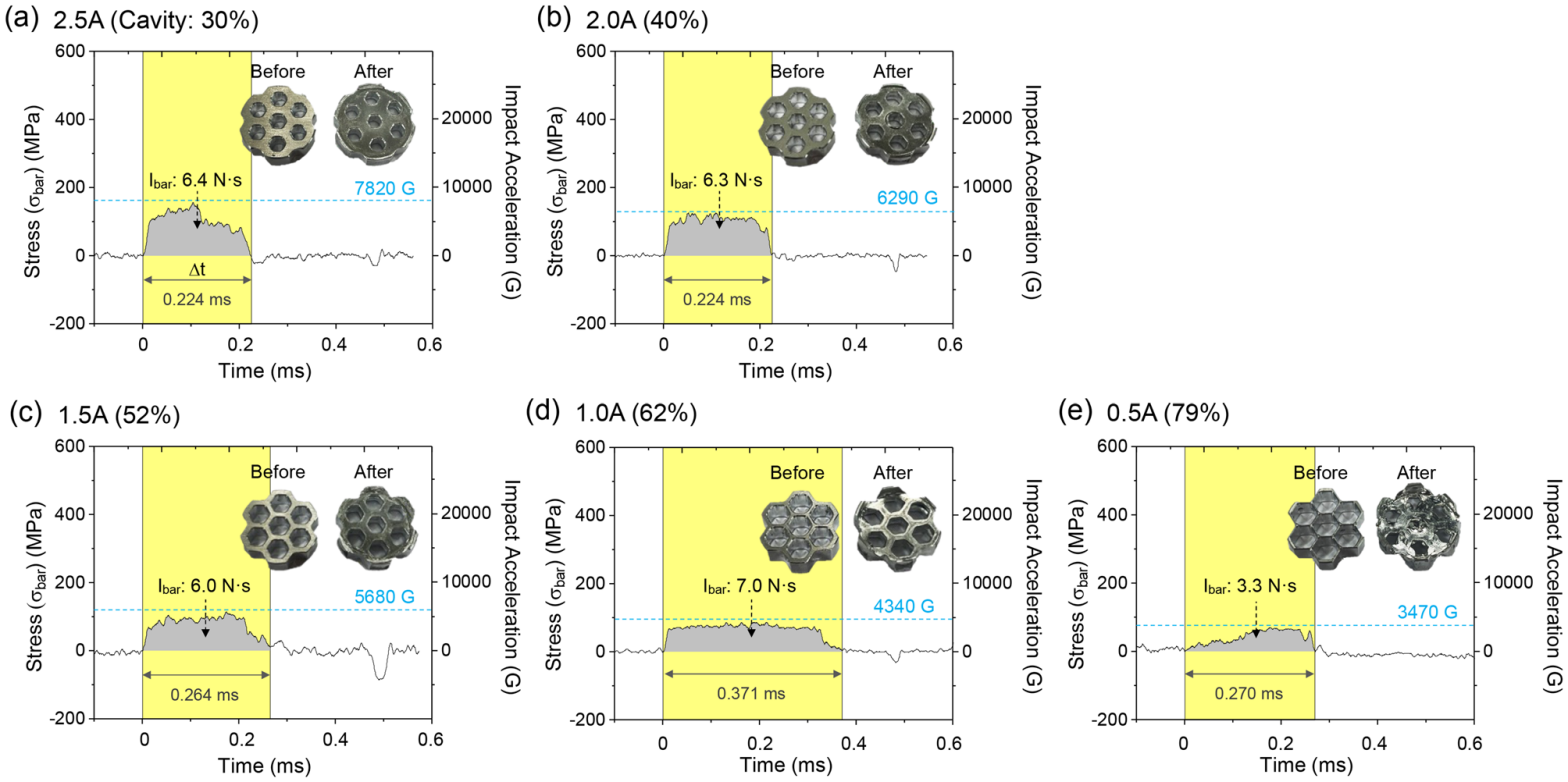
*σ*–t curves of the 6-mm-thick (**a**) 2.5A, (**b**) 2.0A, (**c**) 1.5A, (**d**) 1.0A, and (**e**) 0.5A specimens. Photographs of the specimens before and after the SHPB test are shown within the figures.

Figure 2a–e shows *σ*–t curves of the 6-mm-thick 2.5A, 2.0A, 1.5A, 1.0A, and 0.5A specimens, along with corresponding photographs before and after the SHPB test. The side walls of the transfer bar and incident bar in the SHPB are maintained precisely horizontal even during the test, resulting in the compressive deformation of the specimen. Most of the *σ*–t curves can be divided into three distinct regions: an initial sharp increase, a relatively flat region, and a decreasing segment, which correspond to the elastic deformation stage, crushing stage, and post-failure stage, respectively^16^. The The *σ* of the 2.5A specimen rises to 154 MPa, gradually decreasing to form a broad-peak-shaped curve (Fig. 2a). The *σ* of the 2.0A specimen increases to 112 MPa, stays constant for 0.153 ms, and then decreases, forming a largely-broadened plateau-shaped curve (Fig. 2b). The 1.5A and 1.0A specimens show similar curve shapes to the 2.0A specimen (Fig. 2c,d), with a slight decrease in height and an increase in *Δt*. In the 0.5A specimen, the *σ* increases slowly to 64 MPa, forming a plateau-shaped curve, while both height and *Δt* decreasing (Fig. 2e). Table 1 presents the thickness and reduction ratio compared to the initial thickness. The thickness continuously decreases in the order of the 2.5A, 2.0A, 1.5A, 1.0A, and 0.5A specimens (or as the honeycomb cavity fraction increases: 30, 40, 52, 62, and 79%, respectively), while the specimen diameter expands during dynamic compression.

**Table 1:** Thickness of the specimen center after the SHPB test and the thickness reduction ratio compared to the initial specimen thickness for the 6-mm-thick open-cell Al foam, and honeycomb-structured Al and nylon specimens at the air pressures of 0.3 MPa and 0.5 MPa.

Air pressure (MPa)	Specimen	Cavity fraction (%)	Thickness (mm)	Thickness reduction ratio (%)
0.3	Al Foam	95	0.40 ± 0.04	93.3 ± 0.7
	BA	0	5.50 ± 0.15	8.3 ± 2.5
	2.5A	30	5.01 ± 0.08	16.5 ± 1.3
	2.0A	40	4.86 ± 0.12	19.0 ± 2.0
	1.5A	52	4.35 ± 0.19	27.5 ± 3.2
	1.0A	62	3.41 ± 0.05	43.2 ± 0.8
	0.5A	79	1.11 ± 0.20	81.5 ± 3.3
	BN	0	5.80 ± 0.06	3.3 ± 1.0
	2.5N	30	5.06 ± 0.22	15.7 ± 3.7
	2.0N	40	4.19 ± 0.34	30.2 ± 5.7
	1.5N	52	1.82 ± 0.39	69.7 ± 6.5
	1.0N	62	0.91 ± 0.37	84.8 ± 6.2
0.5	2.0A	40	3.88 ± 0.25	35.3 ± 4.2
	1.0A	62	1.85 ± 0.21	65.7 ± 3.5

Supplementary Figure S1a–d displays *σ*–t curves of the 2.5N, 2.0N, 1.5N, and 1.0N specimens, along with compressed specimen photographs. The *σ* of the 2.5N specimen rises to 72 MPa, gradually decreasing to form a largely-broadened plateau (Fig. S1a). The 2.0N specimen exhibits lower height and longer *Δt*, further broadening the curve (Fig. S1b), which becomes more pronounced in the 1.5N specimen (Fig. S1c). The 1.0N specimen shows a slow increase in *σ* to 86 MPa, forming a plateau-shaped curve with increased height and decreased *Δt* (Fig. S1d). Curve data indicate that broadening occurs as the cavity fraction increases, up to the point where the honeycomb wall withstands the impact. The thickness of the nylon specimen center also continuously decreases with increasing cavity fraction, similar to Al sheet specimens, but the reduction is larger (Table 1).

### Evaluation of impact energy-absorbing performance by defining impact momentum (***I***_***bar***_) and maximum impact acceleration (***a***^***max***^)

To understand the varied *σ*–t curve shapes with cavity fraction and material, we measured the impact momentum applied to the incident bar (*I*_*bar*_) and the maximum impact acceleration of the striker bar (*a*_*sbar*_^*max*^) from Figs. 1 and 2, along with maximum stress (*σ*_*max*_) and incident wave length (*Δt*). The following equation describes the *I*_*bar*_:

$${I}_{bar}= {\sigma }_{bar}\cdot {A}_{bar}\cdot t ({10}^{6} N\cdot s)$$

where A_bar_ is the incident bar area (284 mm^2^). *I*_*bar*_ is determined as the area beneath the *σ*–t curve using an equation. Assuming that the force of the striker bar (*F*_*sbar*_) was identical to the force transferred to the incident bar (*F*_*bar*_), the impact acceleration (*a*_*sbar*_) is addressed by the following equations:

$${a}_{sbar}= \frac{1}{{\rho }_{sbar}\cdot {L}_{sbar}}\cdot \frac{2}{{F}_{gage}}\frac{{E}_{bar}}{{V}_{in}}\cdot {V}_{out} ({\text{m}}/{{\text{s}}}^{2})$$

$$=\frac{1}{9.8}\cdot \frac{1}{{\rho }_{sbar}\cdot {L}_{sbar}}\cdot \frac{2}{{F}_{gage}}\frac{{E}_{bar}}{{V}_{in}}\cdot {V}_{out} ({\text{G}})$$

$${a}_{sbar}={a}_{bar}=a=2522\cdot {10}^{3}\cdot {V}_{out} (m/{s}^{2})=257\cdot {10}^{3}\cdot {V}_{out} (G)$$

where *ρ*_*sbar*_ and *L*_*sbar*_ are the density and length of the striker bar (8.08 × 10^3^ kg/m^3^ and 0.254 m)^13^, respectively. The maximum value of *a*(= *a*_*sbar*_ = *a*_*bar*_) (*a*^*max*^) can be obtained from the maximum output voltage (*V*_*out*_^*max*^).

**Figure 3:**
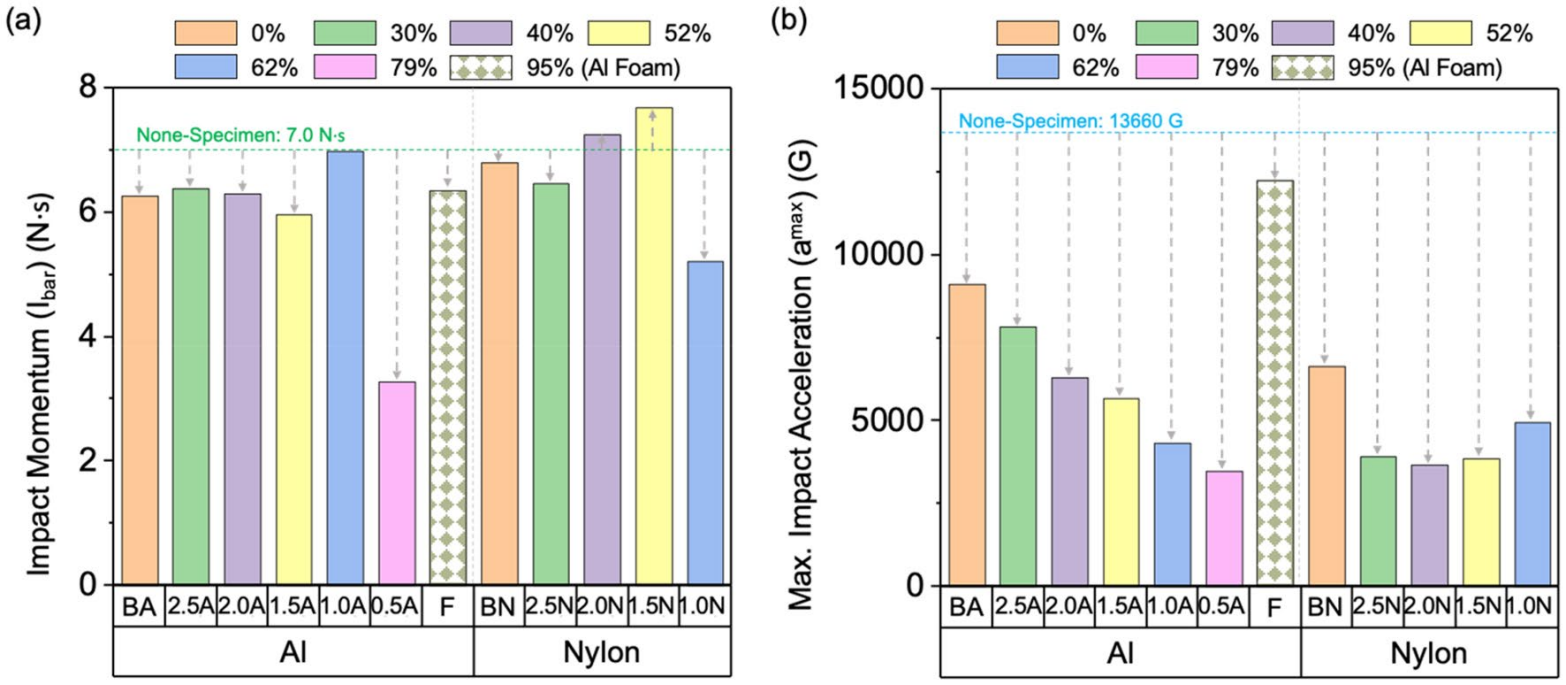
Bar graphs of (**a**) *I*_*bar*_ and (**b**) *a*^*max*^ of the Al and nylon sheet specimens, along with the Al foam specimen and the none-specimen case. The *I*_*bar*_ and *a*^*max*^ of the none-specimen case are marked by the green- and blue-dashed lines, and the *I*_*bar*_ and *a*^*max*^ reduction ratios are indicated by gray-dashed arrows.

Figure 3a and b presents bar graphs of *I*_*bar*_ and *a*^*max*^ for Al and nylon sheet specimens, including the Al foam specimen and the none-specimen case. The none-specimen case exhibits very high *I*_*bar*_ and *a*^*max*^ (7.0 N s and 13,660 G, respectively) indicated by green- and blue-dashed lines. The *I*_*bar*_ and *a*^*max*^ of the Al foam specimen are 6.4 N s and 12,230 G, respectively, lower than the none-specimen case but still substantial. The *I*_*bar*_ is 6.3 N s in the BA specimen, slightly lower than the Al foam specimen (6.4 N s), and the *I*_*bar*_ in the 2.5A, 2.0A, and 1.5A specimens remains at this level (6.0–6.4 N s), abruptly decreasing in the 0.5A specimen (3.3 N s), while the *I*_*bar*_ of the 1.0A specimen (7.0 N s) is similar to the none-specimen case. Overall, *I*_*bar*_ values in the honeycomb-structured nylon specimens show no trend with increasing cavity fraction but are somewhat higher (5.2–7.7 N s) than the Al specimens (3.3–7.0 N s). The *I*_*bar*_ reduction ratio, expressed as a percentage (%), is the reduced ratio of *I*_*bar*_ compared to the none-specimen case (7.0 N s), shown by gray-dashed arrows.

The BA specimen has the *a*^*max*^ of 9100 G, lower than the none-specimen case (13,660 G) (Fig. 3b). The honeycomb-structured Al specimens have much lower *a*^*max*^ (3470–7820 G) than the none-specimen case, showing a continuous decreasing trend with increasing cavity fraction. A similar *a*^*max*^ trend is observed in the honeycomb-structured nylon specimens, though the 1.5N and 1.0N specimens have higher *a*^*max*^ (3850 G and 4940 G, respectively) than the 2.0N specimen (3660 G). The *a*^*max*^ reduction ratio, represented by gray-dashed arrows, is the reduced ratio of *a*^*max*^ compared to the none-specimen case (13,660 G).

## Discussion

### Impact momentum (***I***_***bar***_) and maximum impact acceleration (***a***^***max***^)

The honeycomb-structured Al and nylon specimens alter the original rectangular *σ*–t curve shape of the none-specimen case to broad-peak or shallow-plateau curves as the honeycomb cavity fraction increases (Figs. 1 and 2, S1). Key energy-absorbing parameters can be derived from the measured values of *I*_*bar*_ and *a*^*max*^ based on these curve shapes. *I*_*bar*_ represents the overall incident wave energy from the striker bar, which decreases as the energy is dissipated by the honeycomb cavities. On the other hand, *a*^*max*^ indicates the largest change in impact velocity applied to the specimen and is a crucial parameter for evaluating the energy-absorbing performance, especially in artillery-firing environments where significant *a*^*max*^ reduction is necessary for effective buffering materials^17–19^.

**Figure 4:**
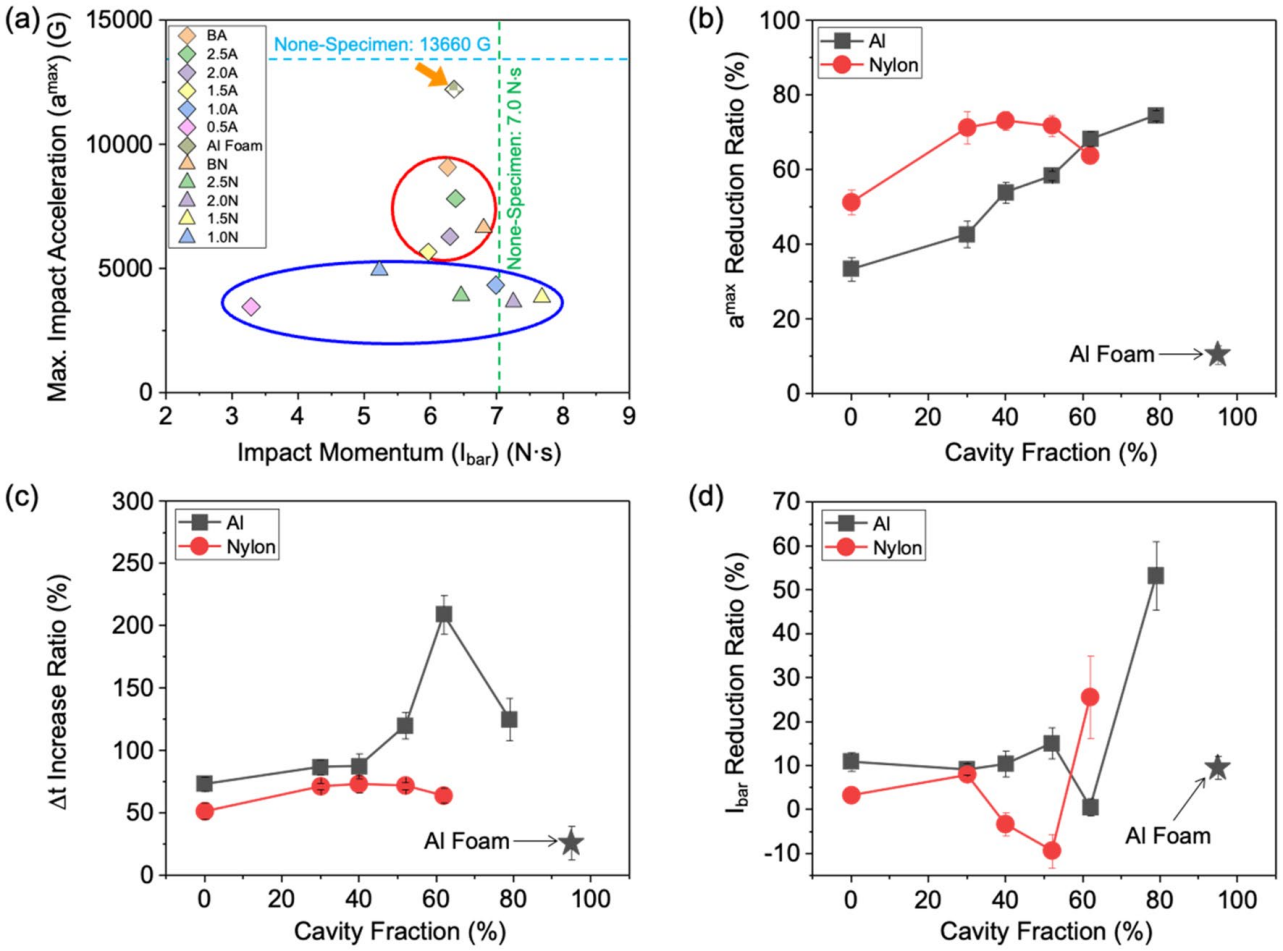
Plot of (**a**) *a*^*max*^ versus *I*_*bar*_ of the honeycomb-structured Al and nylon sheet specimens, along with the Al foam specimen and the none-specimen case, and plots of (**b**) *a*^*max*^ reduction ratio, (**c**) *Δt* increase ratio, and (**d**) *I*_*bar*_ reduction ratio as a function of cavity fraction for the honeycomb-structured Al and nylon specimens.

Figure 4a illustrates *a*^*max*^ plotted against *I*_*bar*_ for honeycomb-structured Al and nylon sheet specimens, as well as the Al foam specimen and the none-specimen case. The *a*^*max*^ and *I*_*bar*_ for the none-specimen case are depicted by blue- and green-dashed lines, respectively. The Al foam specimen lies at the highest data point (orange-arrow mark) due to its notably higher *a*^*max*^ compared to other Al or nylon specimens. Data points of BA, BN, 2.5A, and 2.0A specimens mostly cluster in the upper area (red circle), while those of 1.5A, 1.0A, 0.5A, and 2.0N, 1.5N, and 1.0N specimens are positioned in the lower area (blue ellipse). This suggests a continuous decrease in *a*^*max*^ as the cavity fraction increases, whereas *I*_*bar*_ does not closely follow this decreasing trend.

### Effects of honeycomb-cavity fraction on ***a***^***max***^ and ***I***_***bar***_ reduction ratios

Figure 4b–d displays plots of *a*^*max*^ reduction ratio, *Δt* increase ratio, and *I*_*bar*_ reduction ratio as a function of cavity fraction for the honeycomb-structured Al and nylon specimens. The *a*^*max*^ reduction ratio is the lowest in the BA specimen and gradually increases with increasing cavity fraction in the Al specimens (Fig. 4b). The 1.0A and 0.5A specimens show approximately twice as high *a*^*max*^ reduction ratios as the BA specimen. Comparing the 0.5A specimen’s *a*^*max*^ reduction ratio with the Al foam specimen (indicated by a star-symbol mark) with higher cavity fraction (79 *vs*. 95%), it is about seven times higher than the Al foam specimen’s. The low *a*^*max*^ reduction ratio in the Al foam specimen differs from the general trend observed in the honeycomb-structured Al specimens^3,20,21^, where *a*^*max*^ reduction ratio increases with cavity fraction due to energy absorption rather than pore closure. This makes the high-cavity-fraction honeycomb-structured Al specimens more preferable for effective energy absorption and lightweight buffering materials, particularly in applications like artillery firing where higher energy absorption is desired^22,23^. In the nylon specimens, the *a*^*max*^ reduction ratio increases with increasing cavity fraction until it reaches 40%, following a similar trend to the Al specimens, after which it slightly decreases. The *a*^*max*^ reduction ratios are higher at each cavity fraction in the nylon specimens compared to the Al specimens until 52% cavity fraction. These *a*^*max*^ reduction ratio results suggest that a similar up and down curve pattern may also be evident in the Al specimens. While machining difficulties limited the possibility of fabricating specimens with a honeycomb wall thickness thinner than 0.5 mm, looking at the results for the Al foam specimen with a cavity fraction of 95%, it can be inferred that beyond a 79% cavity fraction, the *a*^*max*^ reduction ratio is likely to decrease. Therefore, as the cavity fraction increases for both Al and nylon specimens, there is an initial increase in *a*^*max*^ reduction ratio up to a certain point. However, if the honeycomb wall thickness becomes too thin, it may not withstand compressive loads, resulting in easy deformation and a reduction in energy absorption capacity.

The *Δt* increase ratio exhibits an up and down pattern as cavity fraction increases, with the Al specimens showing a more pronounced effect compared to the nylon specimens (Fig. 4b). The presence of a similar up and down pattern in both Al and nylon specimens might be considered a precursor, suggesting that the trend in *a*^*max*^ reduction ratio could follow a pattern similar to that observed in the *Δt* increase ratio. Thus, the occurrence of up and down pattern in both Al and nylon specimens indicates that the trend in *Δt* increase ratio closely mirrors the trend observed in the *a*^*max*^ reduction ratio. The *Δt* values in the Al specimens are consistently higher than in the nylon specimens across all cavity fraction ranges.

The *I*_*bar*_ reduction ratio remains relatively stable or decreases slightly until a cavity fraction of about 50–60%, after which it abruptly increases (Fig. 4d). Despite this, the *I*_*bar*_ reduction ratio do not show a clear correlation with the *a*^*max*^ reduction ratio data, which steadily increases (Fig. 4b). The plotted data in Fig. 4b–d indicate that the honeycomb-structured specimens with higher cavity fraction exhibit better energy-absorbing performance based on the *a*^*max*^ reduction ratio data.

### *σ*–t curve-shape analyses

This section analyzes the variation in the *σ*–t curve shape for honeycomb-structured Al and nylon sheet specimens. The curves of the Al specimens continuously broaden, leading to a slight reduction in curve height and *a*^*max*^ (Fig. 2a–e). When the cavity fraction is small (e.g., 30 or 40% in the 2.5A or 2.0A specimen), energy absorption occurs mainly during the closure of interior cavities after the impact (Fig. 2a,b). As the cavity fraction increases, the curves become more broadened, taking on a shallow-plateau shape as the specimen center’s thickness decreases (Fig. 2d,e, Table 1). This results in a significant reduction in *a*^*max*^ (Fig. 3b), with *Δt* also increasing continuously to allow sufficient time for energy absorption. In high-cavity-fraction specimens like 1.0A and 0.5A, effective and continuous energy absorption occurs during the extended *Δt*, with a substantial portion of the impact energy utilized for energy absorption. The 0.5A specimen achieves the highest *a*^*max*^ reduction ratio of 53.3% (Fig. 4b) as effective energy absorption begins early in *Δt* by sufficiently closing the cavities. Shallow-plateau *σ*–t curve shapes, seen in high-cavity-fraction specimens, are desirable for better energy absorption compared to near-rectangular or broad-peak curves.

Similar behavior is observed in the honeycomb-structured nylon sheet specimens, where curves continuously broaden to reduce *a*^*max*^. In low-cavity-fraction 2.5N and 2.0N specimens, sufficient energy absorption occurs during closure of interior cavities due the soft and flexible deformation behavior of nylon (Fig. S1a,b, Table 2), resulting in higher *a*^*max*^ reduction ratios compared to the Al specimens with the same honeycomb-wall thickness (Fig. 4b). However, when the cavity fraction further increases to 62%, the soft honeycomb structure is easily deformed and flattened, rapidly closing the cavities. This reduces the energy-absorption capability and *a*^*max*^ reduction ratio, different from the behavior observed in the Al specimens. The reason behind this is that a significant portion of the impact energy is consumed in the early stage of *Δt* to rapidly close the pores, which contributes less to the energy absorption in the later stages. The trend of the *a*^*max*^ reduction ratio curve for the nylon specimens, increasing until a fraction of 52% and then decreasing (Fig. 4b), can be interpreted based on these findings.

**Table 2:** Maximum stress (*σ*_max_), incident wave length (*Δt*), impact momentum (*I*_*bar*_), maximum impact acceleration (*a*^*max*^), *I*_*bar*_ reduction ratio, and *a*^*max*^ reduction ratio measured at the air pressure of 0.3 MPa for the none-specimen case, 6-mm-thick open-cell Al foam, and 6-mm-thick honeycomb-structured Al and nylon specimens.

Specimen	Maximum stress (*σ*_max_) (MPa)	Incident wave length (*Δt*) (ms)	Impact momentum (*I*_*bar*_) (N s)	Maximum impact acceleration (*a*^*max*^) (G)	*I*_*bar*_ reduction ratio** (%)	*a*^*max*^ reduction ratio*** (%)
None*	275 ± 13	0.120 ± 0.009	7.0 ± 0.3	13660 ± 620	–	–
Al Foam	246 ± 5	0.151 ± 0.016	6.4 ± 1.4	12230 ± 240	9.5	10.5
BA	183 ± 9	0.208 ± 0.016	6.3 ± 0.4	9100 ± 430	10.9	33.4
2.5A	157 ± 10	0.224 ± 0.019	6.4 ± 0.2	7820 ± 490	9.2	42.8
2.0A	127 ± 8	0.225 ± 0.021	6.3 ± 0.1	6290 ± 380	10.4	54.0
1.5A	114 ± 3	0.264 ± 0.034	6.0 ± 0.5	5680 ± 160	15.1	58.4
1.0A	87 ± 5	0.371 ± 0.028	7.0 ± 0.7	4340 ± 270	0.6	68.2
0.5A	70 ± 4	0.270 ± 0.033	3.3 ± 0.6	3470 ± 180	53.3	74.6
BN	134 ± 9	0.286 ± 0.018	6.8 ± 0.3	6650 ± 460	3.21	51.3
2.5N	79 ± 10	0.442 ± 0.032	6.5 ± 0.3	3920 ± 590	7.94	71.3
2.0N	74 ± 8	0.463 ± 0.011	7.3 ± 0.2	3660 ± 350	− 3.28^+^	73.2
1.5N	78 ± 3	0.503 ± 0.024	7.7 ± 0.4	3850 ± 380	− 9.37^+^	71.8
1.0N	99 ± 5	0.302 ± 0.020	5.2 ± 0.5	4940 ± 290	25.65	63.8

*None-specimen case where the test specimen is absent from the deceleration-measuring module.

**Ratio of the reduced amount of *I*_*bar*_ in comparison with the *I*_*bar*_ of the none-specimen case.

***Ratio of the reduced amount of *a*^*max*^ in comparison with the *a*^*max*^ of the none-specimen case.

+Negative *I*_*bar*_ reduction ratio values found when the measured *I*_*bar*_ is higher than that of the none-specimen case.

### Effects of specimen thickness and rotation on energy-absorbing performance

**Figure 5:**
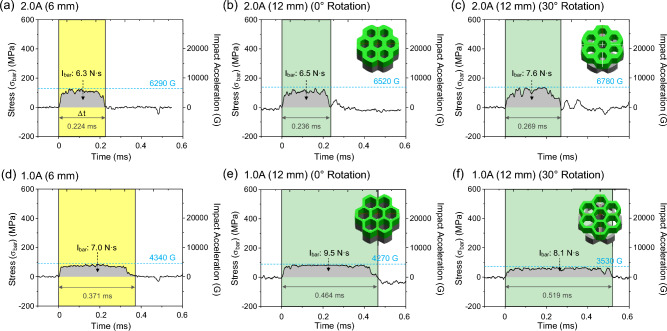
*σ*–t curves of the (**a**) 6-mm-thick 2.0A, (**b**) 12-mm-thick 2.0A, (**c**) 30°-rotated 12-mm-thick 2.0A, (**d**) 6-mm-thick 1.0A, (**e**) 12-mm-thick 1.0A, (**c**) 30°-rotated 12-mm-thick 1.0A specimens. (**a**,**d**) are same to Fig. 4b,d, and the stacking overviews of the 30°-rotated 12-mm-thick specimens are illustrated within (**c**,**f**).

To investigate the impact of specimen thickness on energy-absorbing performance, the 12-mm-thick 2.0A and 1.0A specimens were prepared by stacking two 6-mm-thick sheets at 0 and 30° rotation angles. Figure 5a–f presents *σ*–t curves of the 6- and 12-mm-thick 2.0A and 1.0A specimens, along with the 30°-rotated 12-mm-thick specimens. The 6-mm-thick specimens’ curves (Fig. 5a,d) are identical to those shown in Fig. 2b and d, respectively, and the stacking overviews of the 30°-rotated 12-mm-thick specimens are illustrated in Fig. 5c and f. The 12-mm-thick 2.0A specimen’s curve (Fig. 5b) exhibits a shallow-plateau shape similar to the 6-mm-thick 2.0A specimen (Fig. 5a), with slightly larger curve height and *Δt*. When the upper sheet is rotated at a 30°, the height and *Δt* of the shallow-plateau-shaped curve further increase (Fig. 5c). In the 2.0A specimen, both *I*_*bar*_ and *a*^*max*^ increase with increasing thickness or rotation of the upper sheet.

In the 12-mm-thick 1.0A specimen, the curve also shows a shallow-plateau shape (Fig. 5e), similar to the 6-mm-thick 1.0A specimen (Fig. 5d), but the peak height slightly decreases with increasing thickness, while *Δt* increases. When the upper sheet is rotated at a 30°, the decrease in height and increase in *Δt* become more pronounced (Fig. 5f). In these 1.0A specimens, *a*^*max*^ decreases as the thickness increases or the upper sheet is rotated, while *I*_*bar*_ tends to increase. Considering that the *a*^*max*^ reduction ratio plays a critical role in the assessment of energy absorption capacity, the *a*^*max*^ reduction ratio increases in the 1.0A specimen as thickness increases or the upper sheet is rotated, whereas it decreases in the 2.0 specimen. This difference arises from the variation in honeycomb wall thickness (1.0 mm vs. 2.0 mm) and cavity fraction (62 vs. 40%) between the 2.0A and 1.0A specimens. The *a*^*max*^ reduction ratio increases in thicker or rotated specimens with thinner wall thickness (or higher cavity fraction), such as the 1.0A specimen, where energy absorption occurs continuously and effectively during the extended *Δt* stage. However, the *a*^*max*^ reduction ratio decreases in specimens with thicker wall thickness (or lower cavity fraction), such as the 2.0A specimen. These results further confirm that energy absorption proceeds uniformly and continuously in honeycomb-structured specimens with thinner wall thickness, regardless of specimen thickness and orientation.

Supplementary Figure S2a–d shows photographs of the 12-mm-thick 2.0A and 1.0A specimens before and after the modified SHPB test, along with the measured specimen-center thicknesses. The 1.0A specimens have lower specimen-center thicknesses after the SHPB test compared to the 2.0A specimens due to the higher cavity fraction, and the 30°-rotated specimens have even lower thicknesses. The 30°-rotated 1.0A specimen exhibits a ‘zipper’ mechanism, where the upper and lower sheets adhere together as the honeycomb walls increased cavity fraction and 30° rotation favorably impact energy-absorbing performance as specimen thickness increases.

### Effects of air pressure on energy-absorbing performance

The effects of strain rate on energy-absorbing performance were considered by conducting modified SHPB tests on 6-mm-thick honeycomb-structured 2.0A and 1.0A specimens at air pressures of 0.3 and 0.5 MPa. Comparing the resulting *σ*–t curves (Supplementary Fig. S3a–f), the curve height in the none-specimen case increased from about 275–325 MPa with increased air pressure, maintaining a near-rectangular shape (Fig. S3a,d). In the 2.0A specimen, the curve height decreased and *Δt* increased, resulting in a shallow-plateau shape (Fig. S3e), similar to the result at 0.3 MPa (Fig. S3b). The curve height decreased in the 1.0A specimen (Fig. S3f).

The measured thickness reduction ratios at 0.5 MPa were about 1.5 times higher than at 0.3 MPa (Supplementary Table S1). The *σ*–t data at 0.5 MPa indicated similar wave propagation behavior in the 2.0A and 1.0A specimens compared to 0.3 MPa. The *I*_*bar*_ and *a*^*max*^ values at 0.5 MPa were 10–60% higher than at 0.3 MPa, while the *a*^*max*^ reduction ratio remained similar. As the tendencies of *I*_*bar*_, *a*^*max*^, *I*_*bar*_ reduction ratio, and *a*^*max*^ reduction ratio persisted at the increased air pressure, the evaluation results in ultra-high-strain-rate tests such as artillery-firing or large-scale gas-gun tests would follow those of the present SHPB.

The study focused on honeycomb-structured Al-alloy and MC-nylon sheets and their energy-absorbing performance analyses using the modified SHPB. The method allowed for a quantitative correlation with safety in collisions, impacts, or shocks. While dynamic compressive loading environments present complexities in energy-absorption analyses, the modified SHPB provided valuable insights into energy-buffering concepts and mechanistic interpretations of *a*^*max*^. It offers a promising approach for developing and enhancing honeycomb-structured materials by optimizing capacity, thickness, and orientation. Overall, this study provides a foundation for the design and evaluation of effective honeycomb-structured materials for superior energy absorption in impact scenarios, offering potential applications in diverse field with a focus on safety and performance enhancement.

## Conclusions

This study evaluated the energy-absorbing performance of honeycomb-structured Al-alloy and MC-nylon sheet specimens using a modified split Hopkinson pressure bar (SHPB).

1. The SHPB setup was modified to utilize only the incident wave to prevent or minimize mechanical-energy dissipation during the striker-bar impact. The ‘none-specimen case’ exhibited a near-rectangular *σ*–t curve shape with rapid stress (*σ*) increase to approximately 250 MPa, remaining constant for about 0.10 ms before dropping. When the honeycomb-structured Al-alloy or nylon sheet specimens were inserted, the curve shape changed to broad-peak or shallow-plateau shapes.
2. Key parameter for evaluation were impact momentum (*I*_*bar*_) and maximum impact acceleration (*a*^*max*^). *I*_*bar*_ represented the incident wave’s overall energy, while *a*^*max*^ indicated the largest change in impact velocity on the specimen, particularly critical in artillery-firing environments. The *a*^*max*^ reduction ratio, defined as the decrease in *a*^*max*^ in compared to the none-specimen case, was lowest in bulk Al sheets and gradually increased with higher cavity fractions in the honeycomb-structured Al specimens. The high-cavity-fraction Al specimens offered better energy absorption and lighter-weight buffering materials.
3. In the nylon specimens, the *a*^*max*^ reduction ratio increased with cavity fraction up to 52%, and surpassed that of the Al specimens, then decreased slightly. The low-cavity-fraction nylon specimens absorbed energy effectively during cavity closure due to their soft and flexible nature, resulting in higher *a*^*max*^ reduction ratios. However, in the high-cavity-fraction nylon specimens, the soft honeycomb structure flattened rapidly, reducing the *a*^*max*^ reduction ratio.
4. Thicker honeycomb-structured Al specimens (12 mm) with high cavity fractions were prepared by stacking 6-mm-thick sheets at 0 and 30° rotation angles. Their *σ*–t curves exhibited shallower plateau shapes, with even shallower curves at 30° rotation. As a result, the *a*^*max*^ reduction ratio increased in thicker or rotated specimens due to continuous and sufficient energy absorption, with the applied impact energy utilized by abundant cavity fractions. These findings offer valuable insights for designing and enhancing effective honeycomb-structured materials for superior energy absorption in various applications.

## Method

### Honeycomb-structured Al-alloy or MC-nylon sheets

**Figure 6:**
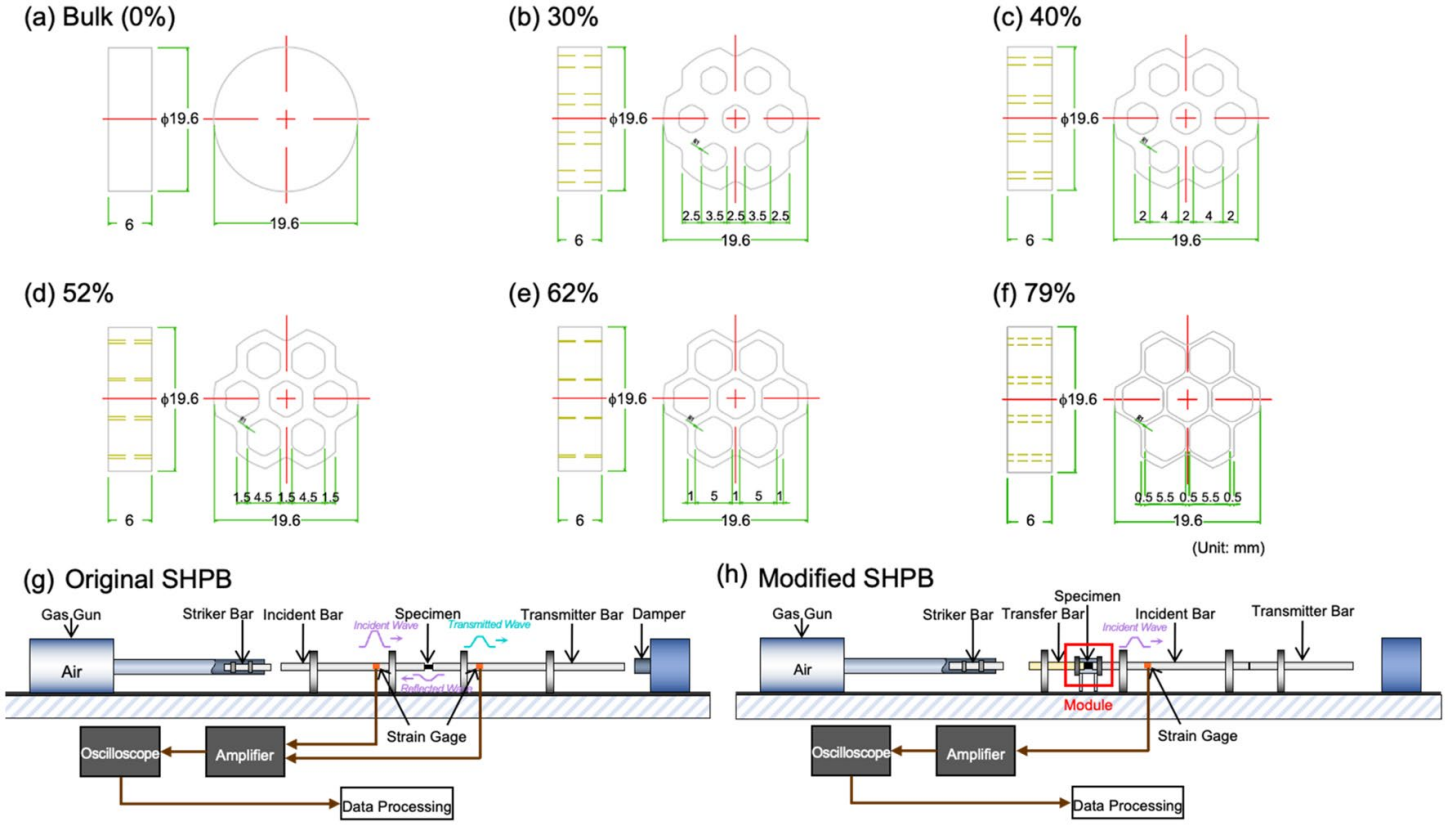
The Al or nylon sheet specimens of (**a**) the bulk Al or nylon sheet specimens without honeycomb cavities are referred to as ‘BA or BN’, respectively, (**b**) 2.5, (**c**) 2.0, (**d**) 1.5, (**e**) 1.0, and (**f**) 0.5 mm in honeycomb-wall thickness are referred to as ‘2.5A or 2.5N’, ‘2.0A or 2.0N’, ‘1.5A or 1.5N’, ‘1.0A or 1.0N’, and ‘0.5A or 0.5N’, respectively, and the bulk Al or nylon sheet specimens without honeycomb cavities are referred to as ‘BA or BN’, respectively. Schematic diagrams of the (**g**) original and (**h**) modified split Hopkinson pressure bar (SHPB).

Cylindrical sheets of 19.6ϕ × 6 mm in size were obtained from an extruded 25ϕ-mm AA6061-T6 Al alloy bar or a mono-cast (MC) nylon bar, and honeycomb cavities were machined inside the sheets. The thickness of the wall between the honeycomb cavities was varied from 2.5 to 0.5 mm to have different volume fractions of honeycomb cavities ranging from 30 to 79%, as shown in Fig. 6a–f. The Al or nylon sheet specimens of 2.5, 2.0, 1.5, 1.0, and 0.5 mm in honeycomb-wall thickness are referred to as ‘2.5A or 2.5N’, ‘2.0A or 2.0N’, ‘1.5A or 1.5N’, ‘1.0A or 1.0N’, and ‘0.5A or 0.5N’, respectively, and the bulk Al or nylon sheet specimens without honeycomb cavities are referred to as ‘BA or BN’, respectively. The 0.5N specimen of 0.5 mm in honeycomb-wall thickness was not obtained because of machining difficulties. The volume fractions of honeycomb cavities of the 2.5A (or 2.5N), 2.0A, 1.5A, 1.0A, and 0.5A specimens are 30, 40, 52, 62, and 79%, respectively. A 19.6ϕ × 6-mm-sized cylindrical sheet of open-cell AA6101-T6 Al alloy foam, ‘Duocel® Aluminum Foam Panel’ (porosity; 95%, compressive strength; 2.53 MPa, tensile strength; 1.24 MPa, elastic modulus; 103 MPa), which is a commercial brand name of ERG Aerospace Corp., Oakland, CA, USA^9,24^, was used for the comparison purposes.

### Split Hopkinson pressure bar (SHPB) testing and its modification

Dynamic compressive tests were conducted on the open-cell Al foam specimen (size: 19.6ϕ × 6 mm) using the SHPB, as schematically shown in Fig. 6g^25–27^. The cylindrical specimen was placed between incident and transmitter bars, and was compressively impacted by a 19ϕ-mm striker bar at an air pressure of 0.3 MPa. Strain gages were attached to the bars to detect incident, transmitted, and reflected waves, which were recorded in an oscilloscope. Details of the SHPB testing were described in previous papers^28–31^. In a typical voltage *vs.* time curve of the open-cell Al foam specimen, the incident and reflected waves exhibited a nearly rectangular shape, while the transmitted wave was not observed because of the rapid closure of pores inside the foam specimen. The recorded wave was rather weak, and we attempted to observe it using semiconductor strain gauges. However, the wave obtained in this case exhibited excessive fluctuations near the voltage zero level, making it difficult to determine a meaningful wave. As a result, mechanical or energetic analyses of energy-absorbing performance could not be conducted using the current SHPB setup.

To prevent or minimize the dissipation of mechanical energy during the striker-bar impact, the SHPB testing setup was modified by utilizing the incident wave alone, as shown in Fig. 6h. The setup sequence is as follows: the specimen housing was installed in front of the incident bar, and the specimen was placed inside the housing. To ensure the specimen maintains its shape within the housing, a closure cap, acting as a transfer bar to transmit waves, was inserted on the opposite side of the incident bar within the housing. Therefore, the setup involved placing the components in the following order: striker bar, transfer bar, specimen, and incident bar. Pulse shapers generally used for minimizing wave distortion were not utilized in this setup. When the striker bar was launched and struck the transfer bar, a compressive wave was transmitted through the transfer bar, specimen, and incident bar. This wave deformed the resistance-based strain gauges attached to the surface of the incident bar, recording the waveform. The transfer bar, having the same dimensions as the striker bar, served the multiple purposes such as holding the specimen, acting as a striking medium, and transferring the incident wave. The incident bar was initially contacted with the transmitter bar, and then they were separated once the wave propagated to the transmitter bar, thereby allowing the detection of only the incident wave at the strain gage.

### Supplementary Information

Supplementary Figures.

Supplementary Table S1.

## Data Availability

The raw/processed data required to reproduce these findings cannot be shared at this time due to technical or time limitations but will be available on reasonable request from the corresponding author.
