# Supplementary material for: Energy-absorption analyses of honeycomb-structured Al-alloy and nylon sheets using modified split Hopkinson pressure bar
Source: Sci Rep. 2023 Dec 18;13:22597. doi: 10.1038/s41598-023-49386-6 (PMC10730563; doi:10.1038/s41598-023-49386-6)
Supplement: Supplementary file 1 — Supplementary Figures. [file 41598_2023_49386_MOESM1_ESM.pptx]

## Slide 1
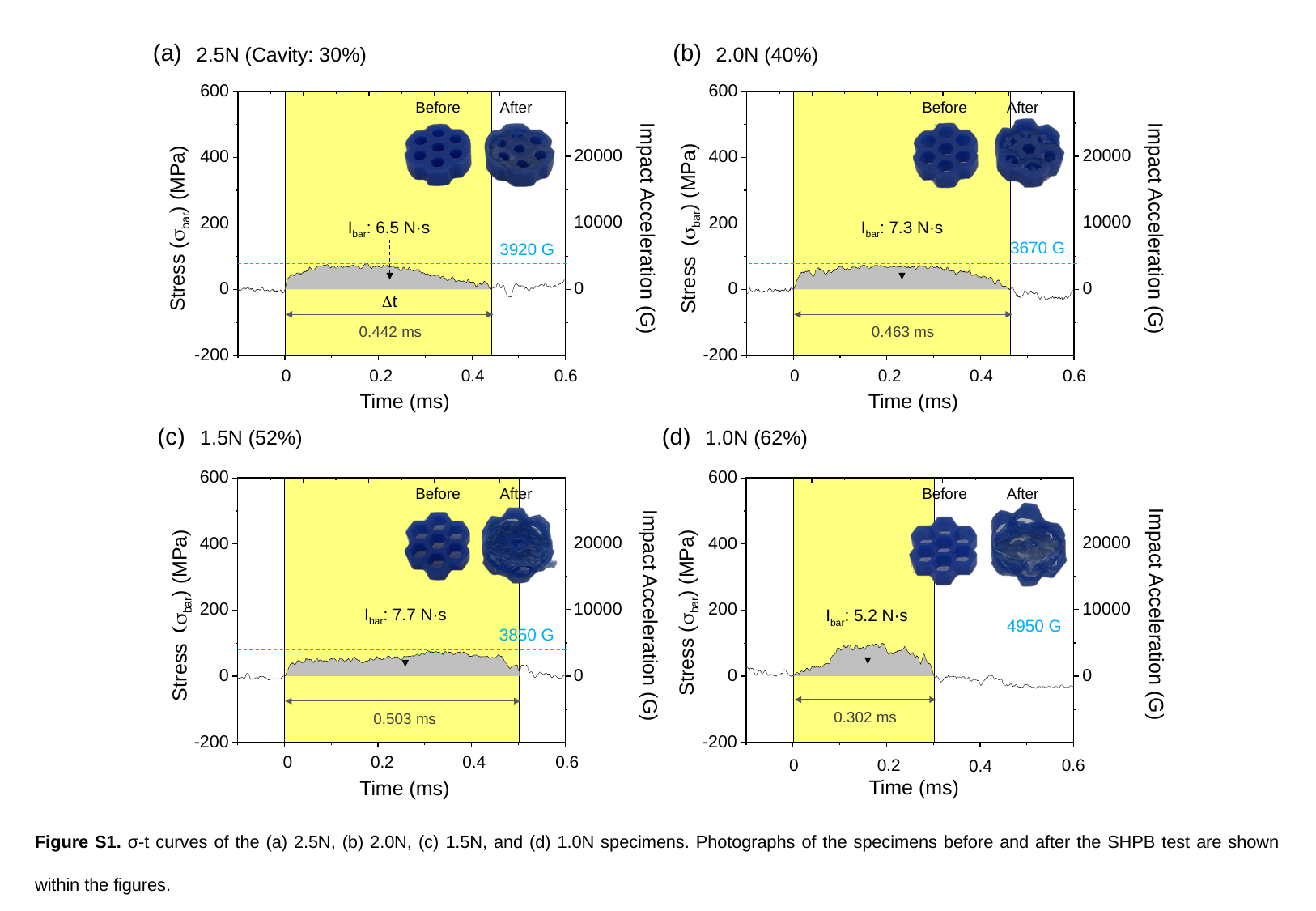

(a)
(b)
2.0N (40%)
2.5N (Cavity: 30%)
Before
After
Before
After
Stress (sbar) (MPa)
Impact Acceleration (G)
Stress (sbar) (MPa)
Impact Acceleration (G)
Ibar: 6.5 N·s
Ibar: 7.3 N·s
3670 G
3920 G
Dt
0.442 ms
0.463 ms
0
0.2
0.6
0.4
0
0.2
0.6
0.4
Time (ms)
Time (ms)
(c)
(d)
1.5N (52%)
1.0N (62%)
Before
After
Before
After
Stress (sbar) (MPa)
Impact Acceleration (G)
Stress (sbar) (MPa)
Impact Acceleration (G)
Ibar: 7.7 N·s
Ibar: 5.2 N·s
4950 G
3850 G
0.302 ms
0.503 ms
0
0.2
0.6
0.4
0
0.2
0.6
0.4
Time (ms)
Time (ms)
Figure S1. σ-t curves of the (a) 2.5N, (b) 2.0N, (c) 1.5N, and (d) 1.0N specimens. Photographs of the specimens before and after the SHPB test are shown within the figures.

## Slide 2
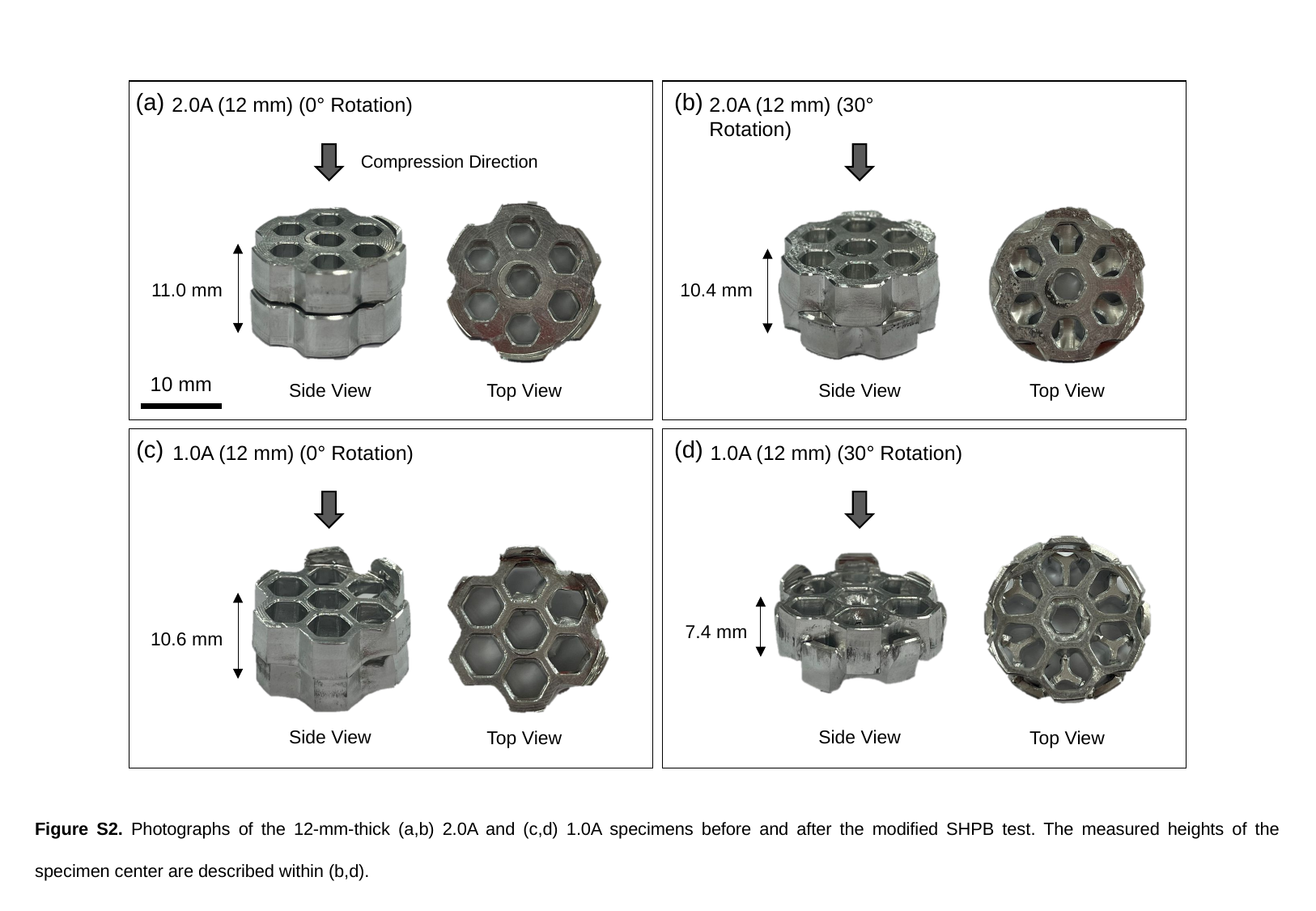

(a)
(b)
2.0A (12 mm) (0° Rotation)
2.0A (12 mm) (30° Rotation)
Compression Direction
11.0 mm
10.4 mm
10 mm
Side View
Top View
Side View
Top View
(c)
(d)
1.0A (12 mm) (0° Rotation)
1.0A (12 mm) (30° Rotation)
7.4 mm
10.6 mm
Side View
Side View
Top View
Top View
Figure S2. Photographs of the 12-mm-thick (a,b) 2.0A and (c,d) 1.0A specimens before and after the modified SHPB test. The measured heights of the specimen center are described within (b,d).

## Slide 3
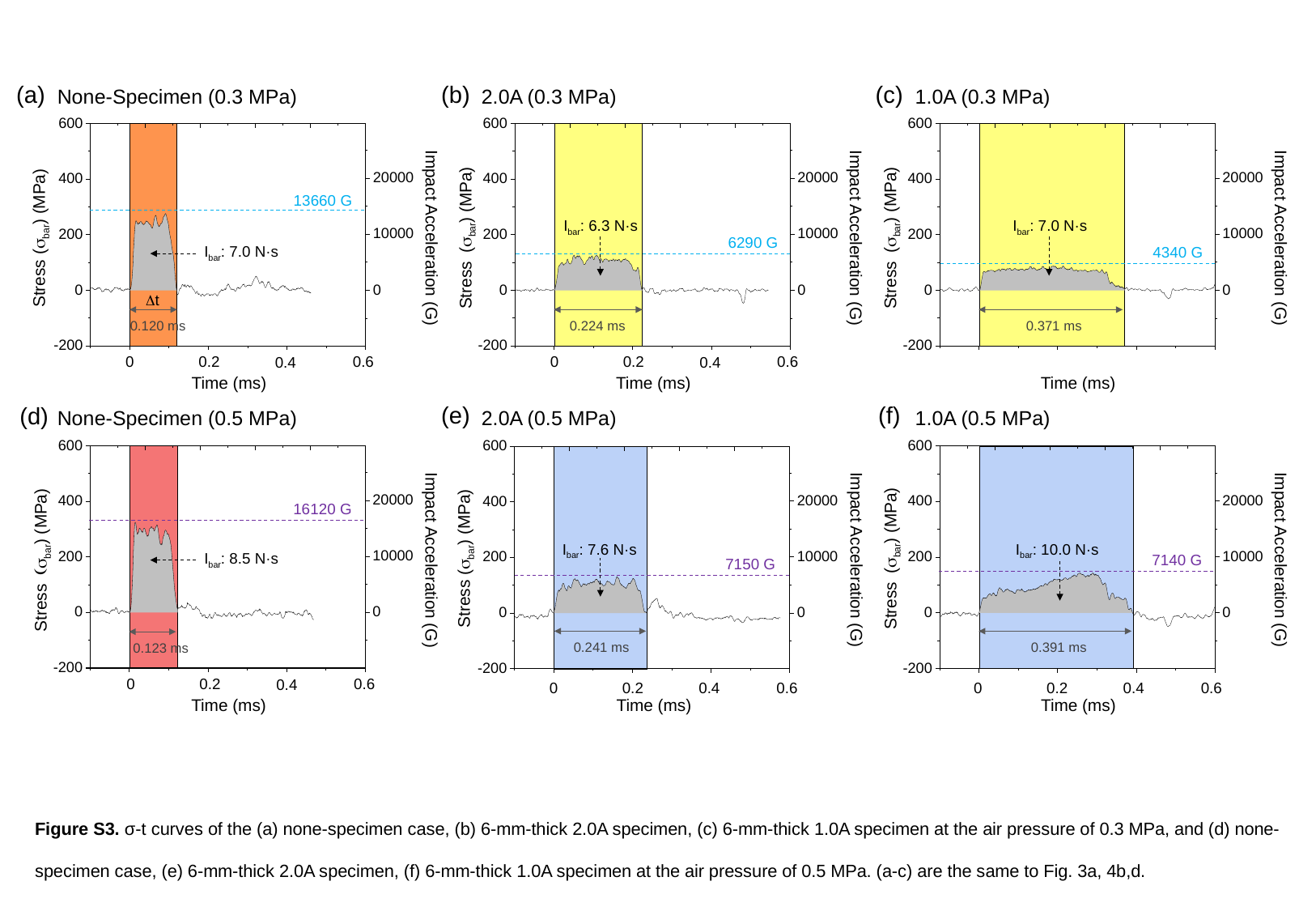

(a)
(b)
(c)
2.0A (0.3 MPa)
1.0A (0.3 MPa)
None-Specimen (0.3 MPa)
13660 G
Ibar: 6.3 N·s
Ibar: 7.0 N·s
Stress (sbar) (MPa)
Impact Acceleration (G)
Stress (sbar) (MPa)
Impact Acceleration (G)
Stress (sbar) (MPa)
Impact Acceleration (G)
6290 G
Ibar: 7.0 N·s
4340 G
Dt
0.120 ms
0.224 ms
0.371 ms
0
0.2
0.6
0.4
0
0.2
0.6
0.4
Time (ms)
Time (ms)
Time (ms)
(e)
(f)
(d)
2.0A (0.5 MPa)
1.0A (0.5 MPa)
None-Specimen (0.5 MPa)
16120 G
Ibar: 7.6 N·s
Ibar: 10.0 N·s
Stress (sbar) (MPa)
Stress (sbar) (MPa)
Ibar: 8.5 N·s
Impact Acceleration (G)
Impact Acceleration (G)
Stress (sbar) (MPa)
Impact Acceleration (G)
7140 G
7150 G
0.241 ms
0.391 ms
0.123 ms
0
0.2
0.6
0.4
0
0.2
0.6
0.4
0
0.2
0.6
0.4
Time (ms)
Time (ms)
Time (ms)
Figure S3. σ-t curves of the (a) none-specimen case, (b) 6-mm-thick 2.0A specimen, (c) 6-mm-thick 1.0A specimen at the air pressure of 0.3 MPa, and (d) none-specimen case, (e) 6-mm-thick 2.0A specimen, (f) 6-mm-thick 1.0A specimen at the air pressure of 0.5 MPa. (a-c) are the same to Fig. 3a, 4b,d.
