# Supplementary material for: Energy-absorption analyses of honeycomb-structured Al-alloy and nylon sheets using modified split Hopkinson pressure bar
Source: Sci Rep. 2023 Dec 18;13:22597. doi: 10.1038/s41598-023-49386-6 (PMC10730563; doi:10.1038/s41598-023-49386-6)
Supplement: Supplementary file 2 — Supplementary Table S1. [file 41598_2023_49386_MOESM2_ESM.docx]

**Supplementary Table S1.** Maximum stress (σ_max_), incident wave length (Δt), impact momentum (I_bar_), maximum impact acceleration (a^max^), I_bar_ reduction ratio, and a^max^ reduction ratio measured at the air pressures of 0.3 and 0.5 MPa for the none-specimen case and the 6-mm-thick honeycomb-structured 2.0A and 1.0A specimens.

| Specimen | Air  Pressure (MPa) | Maximum  Stress  (σ_max_)  (MPa) | Incident  Wave  Length (Δt)  (ms) | Impact Momentum  (I_bar_)  (N∙s) | Maximum  Impact  Acceleration (a^max^) (G) | I_bar_  Reduction  Ratio^**^  (%) | a^max^ Reduction Ratio^***^ (%) |
| --- | --- | --- | --- | --- | --- | --- | --- |
| None^*^ | 0.3 | 275 ± 13 | 0.120 ± 0.009 | 7.0 ± 0.3 | 13660 ± 620 | - | - |
|  | 0.5 | 325 ± 7 | 0.123 ± 0.008 | 8.5 ± 0.5 | 16120 ± 340 | - | - |
| 2.0A | 0.3 | 127 ± 8 | 0.225 ± 0.021 | 6.3 ± 0.1 | 6290 ± 380 | 10.4 | 54.0 |
|  | 0.5 | 144 ± 4 | 0.241 ± 0.022 | 7.6 ± 0.4 | 7150 ± 210 | 10.9 | 55.7 |
| 1.0A | 0.3 | 87 ± 5 | 0.371 ± 0.028 | 7.0 ± 0.7 | 4340 ± 270 | 0.6 | 68.2 |
|  | 0.5 | 144 ± 4 | 0.391 ± 0.037 | 10.0 ± 0.5 | 7140 ± 190 | -17.9^+^ | 55.7 |

*None-specimen case where the test specimen is absent from the deceleration-measuring module.

**Ratio of the reduced amount of I_bar_ in comparison with the I_bar_ of the none-specimen case.

***Ratio of the reduced amount of a^max^ in comparison with the a^max^ of the none-specimen case.

+Negative I_bar_ reduction ratio values found when the measured I_bar_ is higher than that of the none-specimen case.
